# The Pharmacological Activity of *Camellia sinensis* (L.) Kuntze on Metabolic and Endocrine Disorders: A Systematic Review

**DOI:** 10.3390/biom10040603

**Published:** 2020-04-13

**Authors:** Marta Sánchez, Elena González-Burgos, Irene Iglesias, Rafael Lozano, M. Pilar Gómez-Serranillos

**Affiliations:** 1 Department of Pharmacology, Pharmacognosy and Botany, Faculty of Pharmacy, Universidad Complutense de Madrid, Plaza Ramon y Cajal s/n, Ciudad Universitaria, 28040 Madrid, Spain; martas15@ucm.es (M.S.); ireneig@ucm.es (I.I.); pserra@ucm.es (M.P.G.-S.); 2 Department of Chemistry in Pharmaceutical Sciences, Faculty of Pharmacy, Universidad Complutense de Madrid, Plaza Ramon y Cajal s/n, Ciudad Universitaria, 28040 Madrid, Spain; rlozano@ucm.es

**Keywords:** *Camellia sinensis*, metabolic disorders, endocrine disorders, tea

## Abstract

Tea made from *Camellia sinensis* leaves is one of the most consumed beverages worldwide. This systematic review aims to update *Camellia sinensis* pharmacological activity on metabolic and endocrine disorders. Inclusion criteria were preclinical and clinical studies of tea extracts and isolated compounds on osteoporosis, hypertension, diabetes, metabolic syndrome, hypercholesterolemia, and obesity written in English between 2014 and 2019 and published in Pubmed, Science Direct, and Scopus. From a total of 1384 studies, 80 reports met inclusion criteria. Most papers were published in 2015 (29.3%) and 2017 (20.6%), conducted in China (28.75%), US (12.5%), and South Korea (10%) and carried out with extracts (67.5%, especially green tea) and isolated compounds (41.25%, especially epigallocatechin gallate). Most pharmacological studies were *in vitro* and *in vivo* studies focused on diabetes and obesity. Clinical trials, although they have demonstrated promising results, are very limited. Future research should be aimed at providing more clinical evidence on less studied pathologies such as osteoporosis, hypertension, and metabolic syndrome. Given the close relationship among all endocrine disorders, it would be of interest to find a standard dose of tea or their bioactive constituents that would be beneficial for all of them.

## 1. Introduction

The incidence and prevalence of metabolic and endocrine disorders such as obesity and type 2 diabetes mellitus are dramatically increasing due to sedentary lifestyle, food intake, and endocrine disruptors, among others [1,2]. In the year 2030, it is estimated that many of these metabolic and endocrine diseases will be responsible for one of the leading causes of death worldwide [3].

*Camellia sinensis* (L.) Kuntze (Theaceae family) is a tree that mainly grows in tropical and subtropical climates. Tea made from the leaves of *Camellia sinensis* is one of the most consumed beverages in the world. Teas can be classified depending on the degree of fermentation as green tea (unfermented tea), white tea and yellow tea (lightly fermented), oolong tea (semi-fermented tea), black tea (fermented tea), and pu-erh tea (post-fermented tea). Black tea is the most produced and consumed tea worldwide (78% of total tea, especially in Western countries) followed by green tea (20%, especially in China, India, and Japan) and oolong tea (<2%) [4]. Flavanols (primary catechin compounds such as epigallocatechin gallate), flavonols and glycosyl derivatives (i.e., apigenin, myricetin, quercetin, rutin), teaflavins and thearubigins have been identified as main bioactive compounds in the leaves of *Camellia sinensis.* The type and amount of these compounds is determined by the degree of fermentation of the leaves. Epigallocatechin-3-gallate is the major compound in green tea and theaflavins are produced during the processing of black tea, providing the characteristic flavor [5,6,7]. The health benefits of *Camellia sinensis* teas include antioxidant, anti-inflammatory, anti-cancer, cholesterol lowering, and cardiovascular protection properties, among others [4,8].

The present systematic review aims to update the pharmacological activity of *Camellia sinensis* (L.) Kuntze on metabolic and endocrine disorders (osteoporosis, hypertension, diabetes, metabolic syndrome, hypercholesterolemia, and obesity).

## 2. Method

### 2.1. Search Strategy

The systematic review included preclinical and clinical studies of *Camellia sinensis* on endocrine and metabolic disorders. The literature search was conducted using combination of the following keywords “*Camellia sinensis*”, “osteoporosis”, “hypertension”, “diabetes”, “metabolic syndrome”, “hypercholesterolemia”, and “obesity” on Pubmed, Science Direct, and Scopus databases. The search years were from 2014 to 2019.

### 2.2. Inclusion and Exclusion Criteria

The inclusion criteria were preclinical (*in vitro* and *in vivo*) and clinical studies, written in English, focused on the pharmacological activity of isolated compounds and extracts of *Camellia sinensis* on metabolic and endocrine disorders. The excluded criteria were case reports, review articles, conference proceedings, and editorial letters. Moreover, studies involving medicinal plant mixtures, galenic formulations, *Camellia* species different than *Camellia sinensis*, functional foods with tea, and comorbidities associated with endocrine and metabolic diseases were excluded.

The literature research was performed by two independent researchers (E.G.-B. and M.S.) and consisted of an initial identification in the above-mentioned databases, followed by duplicated works elimination and, finally, an exclusion of studies that did not meet inclusion criteria established in this systematic review. The research was verified by a third reviewer (M.P.G.-S.) using a predefined spreadsheet designed by the authors.

## 3. Pharmacological Activity. Description of the Data

**Figure 1:**
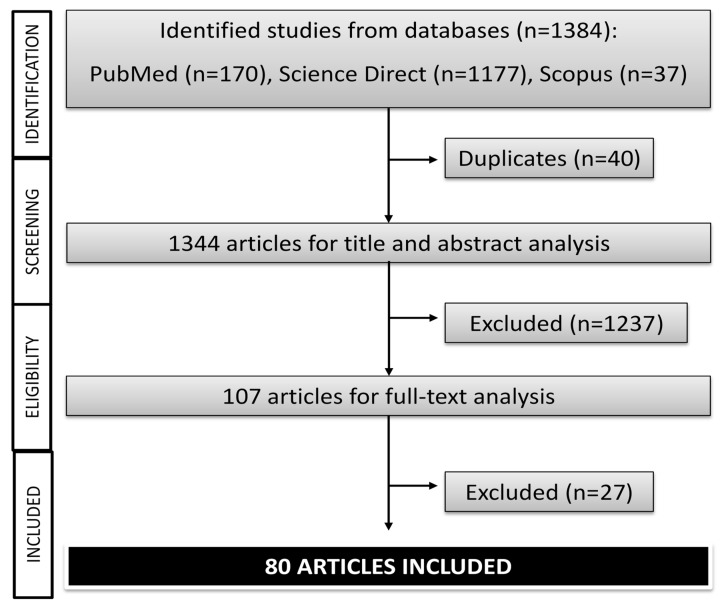
Flowchart of the literature research (*in vitro*, *in vivo*, and clinical trials studies) of *Camellia sinensis*.

Initially, a total of 1384 studies were identified in Pubmed (*n* = 170), Science Direct (*n* = 1177), and Scopus (*n* = 37). However, 40 reports were deleted when appearing in two or more databases (duplicated). Then, 1264 articles were excluded after title and abstract analysis (*n* = 1237) and after full-text analysis (*n* = 27), 80 articles finally being included in this systematic review (Figure 1). Five studies of these 80 reports carried out both *in vitro* and *in vivo* experiments and one study *in vitro* and clinical trials.

**Figure 2:**
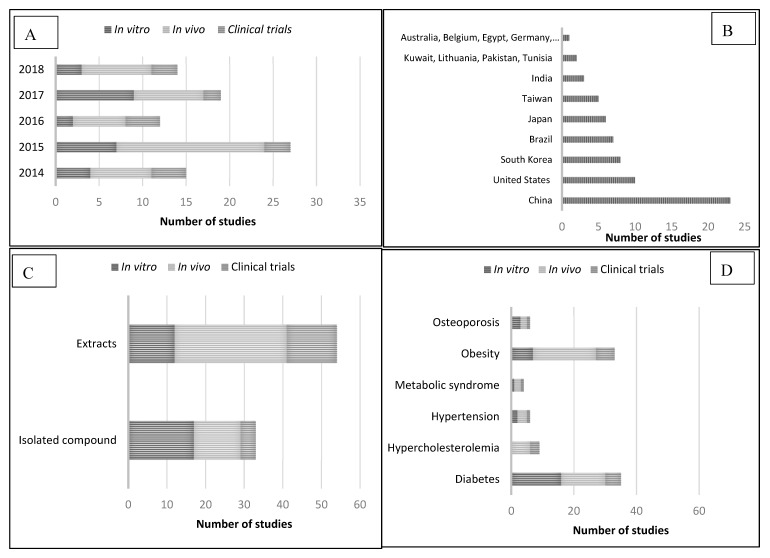
Main characteristics of papers published on pharmacological activity of *Camellia sinensis.* (**A**) Year of publication. (**B**) Research group country. (**C**) Part of the plant used for research. (**D**) Diseases studied in *in vitro*, *in vivo*, and clinical trials studies.

Appendix A (Table A1) and Appendix B (Table A2) include *in vitro* and *in vivo* pharmacological studies, respectively, and these studies were grouped based on disease, extract/isolated compound, experimental model, treatments, major findings, and references. Appendix C (Table A3) includes clinical trials and the main information contained was study (author, year, and country), study design, sample size, population, type of plant, intervention, duration of treatments, and results. Most papers were published in 2015 (*n* = 27, 29.3%) and 2017 (*n* = 19, 20.6%) (Figure 2A). All works included in this systematic review were conducted by research groups of 23 countries, the majority of them from China (*n* = 23, 28.75%), United States (*n* = 10, 12.5%), and South Korea (*n* = 8, 10%) (Figure 2B). These studies were carried out with extracts (*n* = 54, 67.5%) and isolated compounds (*n* = 33, 41.25%) from *Camellia sinensis*. Particularly, extracts were the part of the plant most studied in *in vivo* studies and clinical trials whereas isolated compounds were in the *in vitro* studies. Regarding endocrine and metabolic disorders, diabetes and obesity were the most studies pathologies (*n* = 35 and *n* = 33, respectively) followed by hypercholesterolemia (*n* = 9), osteoporosis (*n* = 6), hypertension (*n* = 5) and metabolic syndrome (*n* = 4). Particularly, diabetes was the most study disease in *in vitro* studies (n = 16) and obesity in *in vivo* studies (*n* = 20), and clinical trials (*n* = 6). In several *in vitro* and *in vivo* studies, the effect of extracts and isolated compounds of *Camellia sinensis* on two different pathologies were studied in the same research work [9,10,11,12,13,14,15,16]. This review is divided into six sections, based on the pathologies, diabetes, hypercholesterolemia, hypertension, metabolic syndrome, obesity, and osteoporosis. Within each pathology, pharmacological activities of tea isolated compounds and extracts was classified in terms of signal transduction, redox system, and changes of biomarkers.

### 3.1. Camellia sinensis and Diabetes

Diabetes mellitus is a chronic metabolic disease that causes abnormally high levels of blood sugar (hyperglycemia) due to a failure in insulin production by pancreas or when the body cannot use insulin effectively [17]. Diabetes mellitus affects about 425 million adults aged between 20 and 79, and it is estimated that in 2025 there will be 629 million. Diabetes mellitus is especially prevalent in low and middle income countries such as those of South-East Asia (82 million) and Western Pacific (159 million) [18]. There are three diabetes mellitus types: type 1, type 2, and gestational. Type 1 diabetes mellitus (insulin-dependent diabetes) is an autoimmune condition that commonly affects individuals during childhood and accounts for around 5% of diabetes mellitus diagnosed cases [17,19]. Type 2 diabetes mellitus (adult onset diabetes) is the most common of the diabetes types (90%–95% of all diagnosed cases worldwide) and it is mainly associated with excess body fat, sedentary lifestyle, and aging [17]. Gestational diabetes mellitus occurs during pregnancy (second or third trimester) because of glucose intolerance; the main risk factors for gestational diabetes mellitus include obesity, ethnicity, age at childbearing, and family history of type 2 diabetes mellitus [20,21].

Most *in vitro* diabetes studies with *Camellia sinensis* are based on the ability of their isolated compounds and extracts to inhibit α-amylase and α-glucosidase activity. In addition, there are several *in vitro* studies with cellular models, mouse 3T3-L1 pre/adipocytes and HepG2 cell lines being the most common. Moreover, for *in vivo* studies, preclinical diabetic animal models (Kunming mice, Sprague-Dawley, and Wistar rats) commonly used to investigate the anti-diabetic properties of tea are streptozotocin and alloxan-induced diabetic animals [10,16,22,23]. Furthermore, the nematode *Caenorhabditis elegans* has been also investigated as diabetic model [24].

Molecular targets in signaling pathways is one of the most successful therapeutic approaches in antidiabetic therapy [25]. Recent studies have determined that tea and its active metabolites can have a therapeutic effect against diabetes through different signaling pathways. Hence, studies on rat islet RIN-5F cell tumor demonstrated that a type II arabinogalactan (200 µg/mL) isolated from green tea leaves increased glucose-stimulated insulin secretion targeting cAMP/PKA [26]. This cAMP/PKA signaling pathway plays a key role in the regulation of glucose homeostasis through gluconeogenesis process and glycogen synthesis and breakdown [25]. Another signal transduction pathway on which tea polysaccharides from green tea (200, 400, and 800 mg/kg b.w. per day for 4 weeks) have been shown to act is the PI3K/Akt signal pathway which stimulates GLUT 4 translocation and activation [10]. Moreover, epigallocatechin gallate promoted glucose uptake by increasing GLUT4 translocation via PI3K/AKT in L6 skeletal muscle cells [15]. Another anti-hyperglycemic strategy is to inhibit sodium glucose transporters such as intestinal SGLT1 and renal SGLT2 that are involved in glucose absorption and to promote GLUT2 and GLUT 4 transporters which facilitate glucose movement across membranes. The acute administration (30 min) and chronic administration (6 weeks) of green tea decoction (50 g/L) and a combination of 4 mg epigallocatechin gallate (EGCG) and 2 mg epigallocatechin (EGC) inhibited SGLT-1 activity and increased *GLUT2* mRNA levels in the jejunum mucosa and *GLUT4* mRNA levels in adipose tissue in Wistar rats fed a high fat diet [12]. Moreover, epigallocatechin gallate inhibited GLUT4-dependent insulin-like growth factor I and II, and stimulated glucose transport in 3T3-L1 adipocytes [27]. Another antidiabetic mechanism has been shown for tea polypeptides from green tea (1000 mg/kg bw/day, p.o. for 5 weeks) which reduced blood glucose and ameliorated diabetic nephropathy in a streptozocin-induced mice model by stimulating the AGEs/RAGE/TGF-β1 signaling pathway and inhibiting the NF-κB pathway [28]. Moreover, pu-erh tea ameliorated insulin resistance by inhibiting IL-6 induction via signal transducer and activator of transcription 3 (STAT3) in C57BL/6J mice [9]. Finally, Chen et al. (2019) found that non-catechin flavonoids (500, 1000, 2000 ppm, for 72 h) ameliorated TNF-α induced insulin resistance by stimulating glucose uptake and inhibiting p38 and JNK pathways in HepG2 cells [29].

The ability of different types of teas and their bioactive compounds to inhibit the enzymes α-amylase and α-glucosidase has been extensively studied in recent years. The enzyme α-amylase, found in saliva and pancreas, catalyzes the hydrolysis of alpha 1–4 bonds of glycogen and starch to form simple sugars (oligosaccharides and disaccharides). Then, α-glucosidase enzyme catalyzes alpha 1–4 bonds of oligosaccharides and disaccharides to form glucose in the small intestine. Both enzymes are a therapeutic target for diabetes mellitus treatment [30]. Yang and Kong (2016) [31] investigated the α-glucosidase inhibitory activity of green tea, black tea, and oolong tea, oolong tea having the lowest IC_50_ value (1.38 µg/mL). Moreover, Oh et al. (2015) [32] compared α-glucosidase inhibitory activity of tea water extracts and tea pomace extracts obtained from green, oolong, and black tea; this research demonstrated that there were no differences between tea water extracts and tea pomace extracts and that green tea was the most active of all assayed type teas (IC_50_ = 2040 µg/mL for tea water extracts and IC_50_ = 1950 µg/mL for tea pomace extracts). Furthermore, the aqueous extract of black tea leaves inhibited α-glucosidase enzyme activity (IC_50_ = 2400 µg/mL for sucrose and IC_50_ = 2800 µg/mL for maltase) but not α-amylase activity [33]. Additionally, black and green teas inhibited α-amylase activity with IC_50_ = 589.86 μg/mL and IC_50_ = 947.80 μg/mL, respectively, and α-glucosidase activity with IC_50_ = 72.31 μg/mL and IC_50_ = 100.23 μg/mL, respectively. Differing chemical composition of these three teas may explain, at least in part, their different effects on diabetes-related enzyme activity. Oolong tea stands out for having dimeric flavan-3-ols (theasinensins), green tea has epigallocatechin-3-gallate as major catechin, and black tea is rich in theaflavins and thearubigins [34,35]. Moreover, the differences in activity for the same type of tea may be due to the fact that the chemical composition is highly influenced by the nature of the green shoots and the procedures to manufacture tea in the producing countries [36]. Apart from studies on black, green, and oolong tea, different ages of pu-erh tea polysaccharide have demonstrated inhibition of α-glucosidase activity, specially 3-year old and 5-year old tea (IC_50_ = 0.583 and 0.438 μg/mL, respectively), however no inhibitory activity was found against α-amylase [37]. In a similar work, Xu et al. (2014) [38] found that pu-erh tea polysaccharides with aging for 3 years and 5 years resulted in inhibition of α-glucosidase enzyme activity with same potency as acarbose (3 years aging) and three times more potently than acarbose (5 years aging). Besides, water extract of pu-erh tea moderately inhibited sucrose activity (IC_50_ = 14.4 μg/mL) and maltase (IC_50_ = 11.4 μg/mL), the compound epigallo-catechin-3-*O*-gallate having the greatest inhibitory activity with IC_50_ = 32.5 μM against sucrose and IC_50_ = 1.3 μM against maltase [14]. In another study, the ethyl acetate fraction from Qingzhuan tea extracts showed significant α-glucosidase inhibitory potential (IC_50 =_ 0.26 μg/mL), attributing this activity to the compounds epigallocatechin gallate and epicatechin gallate. Epicatechin gallate has shown to inhibit α-amylase activity (IC_50_ = 45.30 μg/mL) and α-glucosidase activity (IC_50_ = 4.03 μg/mL) and epigallocatechin gallate inhibited α-glucosidase with IC_50_ = 19.5 μM [15,39]. Moreover, the isolated compound amelliaone A from YingDe black tea inhibited more potently α-glucosidase enzyme activity (IC_50_ = 10.2 μM) than the reference compound acarbose (IC_50_ = 18.2 μM) [40]. Furthermore, Hua et al. [41] investigated the inhibitory activity of flavone and flavone glycosides of green tea (Lu’an GuaPian) on α-glucosidase and α-amylase enzymes; 7 kaempferol monoglycoside was the most active against α-glucosidase (IC_50_ = 40.02 µM) and kaempferol diglycoside against α-amylase (IC_50_ = 0.09 µM). Based on IC_50_ values of the isolated compounds, epigallocatechin gallate and 7 kaempferol monoglycoside resulted as the most promising α-glucosidase inhibitory agents and kaempferol diglycoside the most interesting α-amylase inhibitor.

Oxidative stress (reactive oxygen species/antioxidant imbalance) contributes to the development of diabetes mellitus and its associated complications. Black tea aqueous extract (2.5%) reduced lipid peroxidation levels and increased GSH content in diabetic rats [22]. Moreover, tea polysaccharides from green tea (200, 400, and 800 mg/kg b.w. per day for 4 weeks) increased superoxide dismutase (SOD) and glutathione peroxidase (GPX) activities in diabetic Kunming mice [10]. Furthermore, in another study epigallocatechin-3-gallate demonstrated reduction of lipid peroxidation, protein oxidation, and superoxide level and increased antioxidant enzymatic activity and GSH content in diabetic rats [23].

Moreover, several studies have identified changes in relevant biomarkers for diabetes mellitus after tea extract supplementation. Hence, epigallocatechin-3-gallate (2 mg/kg, p.o., alternative days, 1 month) reduced glucose levels and glycosylated hemoglobin and increased insulin [23]. Moreover, green tea powder (10%) and ethanolic extract of green tea (5%) for 8 weeks reduced glucose levels in Sprague-Dawley rats [16]. Furthermore, green tea extract and pu-erh tea extract (both at doses of 0.8 g/kg with a content of 30% catechin and 10% caffeine) but not epigallocatechin-3-gallate (at a dose of 0.24 g/kg) reduced blood glucose levels in BALB/c mice which suggests that caffeine is essential in the hypoglycemic effect of tea [41]. The doses and time treatments could explain the differences in the effectiveness of epigallocatechin-3-gallate [23,41]. Finally, both black and green teas suppressed the increased production of advanced glycosylation end products in 3T3-L1 preadipocytes [42].

Clinical trials were randomized, double-blind, and placebo-controlled and they evaluated the hypoglycemic effect of green tea (mainly) and black tea. Most of these works included patients of both sexes (except one with overweight women) and aged between 30 and 80 years. The duration of the treatments varied from weeks to months and the doses/day administered were also different in each clinical trial (i.e., 1 g/day; 2.5 g/three times day; 560 mg tea polyphenols/two times day; 200 mg tea extract/day). The parameters measured were different, being analyzed from biochemical parameters such as blood glucose levels to oxidative stress markers. Doses of 1 g of dry extract of green tea and 2.5 g/three times day of black tea for 12 weeks were effective to improve glycemic control even better than the reference drug metformin [43,44]. Moreover, both 560 mg tea polyphenols/two times day for 20 weeks and 200 mg tea extract/day for 9–18 months had an antioxidant effect as evidenced in an increase of superoxide dismutase activity and a decrease of lipid peroxidation [45,46].

### 3.2. Camellia sinensis and Hypercholesterolemia

Hypercholesterolemia (blood cholesterol values > 200 mg/dL) affects over 39% of people worldwide, Europe and America being the most affected continents [44].

Green tea extracts have demonstrated in *in vivo* studies reduced total cholesterol, LDL, and tryglicerides [13,16,47] which is mainly attributed to epigallocatechin gallate and flavonols [48,49]. Moreover, Chungtaejeon aqueous extracts, which is a Korean fermented tea, has shown to decrease hepatic cholesterol, total serum cholesterol, and LDL cholesterol in high fat atherogenic Wistar rats [50].

Clinical trials on the anti-hypercholesterolemia action of black tea and green tea were investigated in patients with high cholesterol levels in randomized, double-bind, and placebo studies. The cholesterol-lowering effect of tea extracts was evaluated by measuring biochemical parameters (i.e., LDL content and total cholesterol) and antioxidant content. Both clinical studies with black tea demonstrated its effectiveness of reducing LDL/HDL ratio, total cholesterol, apolipoprotein B, and oxidative stress. In one of these clinical trials, the effective dose was 2.5 g black tea and phytosterol mixture which contains 1 g plant sterols for 4 weeks [51]. However, for the other study, a specific dose is not specific, but five cups of black tea per day for two 4-week treatment periods [52]. On the other hand, the consumption of “Benifuuki” green tea, which is rich in methylated catechins (3 g of green tea extract/three times daily for 12 weeks) contributed significantly to reduce serum total cholesterol and serum LDL cholesterol compared to “Yabukita” green tea or barley infusion (placebo tea) consumers [53].

### 3.3. Camellia sinensis and Hypertension

Hypertension (blood pressure of ≥ 130/85 mm Hg) is one of the most common cardiovascular diseases which affects around 1.13 billion people worldwide. Endocrine hypertension occurs when there is a hormone imbalance as example in Cushing syndrome, primary aldosteronism, and pheochromocytoma [54,55].

Angiotensin I-Converting Enzyme converts angiotensin I into angiotensin II (vasoconstrictor properties). Infusions and decoctions of four black tea samples (Doors tea, Siliguri tea, Guwahati tea, and Nilgiri tea) (15 μg/mL) were investigated for their ability to inhibit angiotensin I converting enzyme. In general, decoctions were more active than infusions and Nilgiri tea showed the highest inhibitory activity. Antihypertension properties are mainly attributed to thearubigin and theaflavin [56,57]. In another *in vitro* study, pretreatments with black tea extract (0.3–5 μg/mL) and theaflavin-3,3’-digallate (0.03–0.5 μg/mL) for 30 min improved endothelium dependent relaxations in homocysteine (endoplasmic reticulum stress inductor) treated cultured rat aortic endothelial cells [58]. Moreover, San Cheang et al. (2015) [58] also investigated the effect of black tea extract (15 mg/kg/day for 2 weeks) in a rat model of angiotensin II. This study revealed that black tea extract prevented elevated plasma homocysteine levels and downregulated endoplasmic reticulum stress markers. Furthermore, Nomura et al. (2017) [59] investigated the protective effect of three different cultivars of *Camellia sinensis* ("Yabukita", "Sofu" and "Sunrouge") in a model of hypertensive rats fed with a high salt diet. All these tea cultivars reduced urinary NO metabolite and, moreover, "Yabukita" and "Sofu" increased soluble guanylate cyclase expression.

Finally, a single clinical trial has been identified in which the effect of tea, compared with coffee, on blood pressure was evaluated. This study (1352 subjects aged 18–69 years) stratified population in three groups (non-consumers, ≤3 dL/d consumers, and >3 dL/d consumers of tea or coffee). Results showed that consumption of 1 dL/day of tea was associated with lower systolic blood pressure (by 0.6 mm Hg) and lower pulse pressure (by 0.5 mm Hg) [60].

### 3.4. Camellia sinensis and Metabolic Syndrome

The metabolic syndrome is a cluster of metabolic disorders (obesity, hypertension, hypercholesterolemia, and diabetes) that favor cardiovascular disease development [61,62]. It is estimated that around a billion people worldwide suffer from metabolic syndrome [63].

Yang et al. (2014) [64] demonstrated that green tea extract (0.2%–0.5%, w/v) inhibited lipid accumulation during adipogenesis in 3T3-L1 preadipocytes by reducing expression of transcription factors C/EBPα and PPARγ.

The atypical antipsychotic drug olanzapine is associated with severe metabolic side effects through H1 receptor antagonism, 5-HT2 C receptor antagonism, D2 receptor antagonism, and muscarinic (M3) receptor antagonism [65,66]. Green tea aqueous extract (25, 50, and 100 mg/kg/day for 11 days) showed to reduce body weight gain, hypertension, and hyperleptinemia, to decrease blood glucose, triglycerides, total cholesterol, and LDL, and to increase HDL in adult male Wistar rats olanzapine induced [67]. In another *in vivo* study, Xu et al. (2018) [68] investigated the effect of large yellow tea manufactured in the Anhui Province of China on metabolic syndrome in high fat diet treated C57BL/6 mice. This work revealed that yellow tea improved metabolic abnormalities (changes in lipid profile, hyperglycemia, and body weight).

In a clinical trial, patients with metabolic syndrome who received decaffeinated green tea extracts capsules (500 mg green tea extract providing 400 mg catechins; two capsules/time/day for 12 weeks) had lower adiponectin and visfatin concentration levels than control patients who received water [64].

### 3.5. Camellia sinensis and Obesity

Obesity (body mass index ≥ 30) and overweight (body mass index ≥ 25) are increasing due to an augmented intake of energy-dense foods and sedentary lifestyle; it affects more than 1.9 billion adults worldwide. This disease causes about 4 million deaths globally [69] and it is related to other prevalent pathologies including hypertension, type 2 diabetes mellitus, stroke, obstructive sleep apnea, and several cancers [70].

The mouse adipocyte 3T3-L1 cell line has been extensively used to study the *in vitro* effect of tea extracts and its isolated compounds [71,72,73]. In obesity *in vivo* studies, C57BL/6J mice are the most widely animal model since they are susceptible to high fat diet-induced obesity [74,75]. Moreover, other experimental animal models have been used to investigate the effects of different kind of teas and isolated compounds on obesity including Wistar rats, Sprague-Dawley rats, and Swiss mice fed with a high fat diet [76,77].

Several signaling pathways associated with obesity development have been described. The cAMP/PKA pathway participates in adipogenesis process regulation. The stimulation of the serine/threonine kinase protein kinase A (PKA) activity inhibits adipogenesis whereas the inhibition of PKA activity favors the adipogenic process [78]. Green tea polyphenols (epigallocatechin gallate, epigallocatechin, epicatechin gallate, epicatechin, gallocatechin, catechin, and gallocatechin gallate) increased norepinephrine-induced lipolysis via protein kinase A-dependent pathway in the differentiated mouse adipocyte 3T3-L1 cell line [71]. Moreover, the mitogen activated protein kinases (MAPKs) signaling pathways (extracellular signal regulated kinases (ERKs), Jun amino terminal kinases (JNKs), and stress activated protein kinases (p38/SAPKs)) are involved in adipocyte differentiation and in adipogenesis regulation [79]. Green tea polyphenols, gallocatechin gallate, and epigallocatechin-3-gallate decreased MAPK pathway activation as evidenced in downregulation of adipogenic factor expression (CCAAT element binding protein α (C/EBPα), peroxisome proliferator-activated receptor gamma (PPARγ), and sterol regulatory element-binding protein-1c (SREBP-1c)) in a differentiated mouse adipocyte 3T3-L1 cell line [72,73]. Furthermore, the compound gallocatechin gallate has also demonstrated inhibition of inflammation through NF-κB signaling; adipose tissue inflammation is involved in several pathologies associated with obesity such as diabetes mellitus type 2 [73].

In addition to acting on these signaling pathways, several *in vitro* studies focused on the inhibitory effect on the digestive enzyme pancreatic lipase which hydrolyzes triglycerides into monoglycerides and free fatty acids. The ethanol extract of *Camellia sinensis* has demonstrated to suppress lipase activity (IC_50_ = 0.5 mg/mL) [80] and among isolated compounds, highlight in order of pharmacological potency theaflavin-3,3′-digallate (IC_50_ = 1.9 μM), theaflavin-3-gallate (IC_50_ = 3.0 μM), theaflavin-3′-gallate (IC_50_ = 4.2 μM), theaflavin (IC_50_ > 10 μM), epigallo-catechin-3-*O*-gallate (IC_50_ = 13.3 μM), and catechin-3-*O*-gallate (IC_50_ = 13.6 μM) [81,82].

Oxidative stress plays a key role in the development of comorbidities (insulin resistance, cardiovascular problems, diabetes) associated with obesity. The compounds gallocatechin gallate and epigallocatechin-3-gallate at a concentration of 10 μg/mL act as potent antioxidant by decreasing ROS production [73].

Moreover, using experimental *in vivo* models, the effect of tea extracts and their bioactive compounds on changes in relevant biomarkers for obesity have been demonstrated. Green tea extract supplementation has anti-obesity effects by reducing body weight, white adipose tissue fat, liver fat accumulation, and serum triglyceride levels and by increasing lysophospholipids levels and energy expenditure [83,84,85,86,87,88,89,90,91]. These anti-obesity properties of green tea are mainly attributed to its polyphenols (i.e., epigallocatechin gallate and epigallocatechin) and polysaccharides [12,15]. Moreover, Heber et al. (2014) [74] compared the effects of decaffeinated polyphenol extracts from green tea, black tea, and oolong tea in in male C57BL/6J mice fed a high fat diet. This study revealed that all teas reduced body weight, total visceral fat volume, and liver lipid weight and, that green tea polyphenols and oolong tea polyphenols also increased adiponectin gene expression. Besides, oolong tea reduced body weight and fat accumulation by regulating fatty acid oxidation and energy expenditure [75]. Moreover, oolong tea, white tea, yellow tea, pu-erh tea, and black tea have also showed to decrease body weight, plasma triglycerides, white fat accumulation, and fatty acid synthesis and to increase energy expenditure, fatty acid oxidation, and fecal triglycerides excretion in Wistar rats, Sprague-Dawley rats and Swiss mice fed with a high fat diet [76,77]. Furthermore, other studies showed that pu-erh tea and Traditional Korean Chungtaejeon, which are fermented teas, possess anti-obesity properties by ameliorating hepatic lipid metabolism and decreasing fat mass [9,92]. Studies on bioactive compounds isolated from tea extracts have shown that epigallocatechin-3-gallate is responsible of reducing body weight, fat infiltration in liver tissue, and of increasing serum lipid profiles [11,93] and teasaponin reduced body weight gain and improved gut microbiota alteration and cognitive decline in obese C57BL/6J mice [14].

Clinical studies evaluate the anti-obesity activity of green tea and its main secondary metabolite epigallocatechin-3-gallate. These clinical trials were mainly randomized, double-blind and placebo-controlled, except one which was single-blind and another one which was longitudinal. The duration of these clinical trials was mainly weeks (6, 8, and 12 weeks), although there is a study with 12-month intervention. There is also variability in the administered doses from 300 mg/day of EGCG to 856.8 mg/day. Analyzing the different clinical trials, it has been observed that high doses of EGCG (856.8 mg for 6–12 weeks) do have anti-obesity activity, reducing weight in women and men with a body mass index (BMI) ≥ 27 kg/m^2^ [94,95]. However, in another study in which a dose of 843 mg of EGCG was administered for 12 months, it had no effect on reductions in adiposity nor body mass index. Compared with the previous cited studies, this lack of efficacy can be attributed to the fact that this last study has only been performed with postmenopausal obese women [96]. On the other hand, low doses of EGCG (300 mg/day and 560 g/day for 12 weeks) do not have any beneficial effect on weight control [97,98]. Finally, another clinical trial elucidated that the possible mechanism of action by which EGCG exerts its anti-obesity activity is by increasing HIF1-alpha (HIF1-α) and rapamycin-insensitive companion of mTOR (RICTOR) [99].

### 3.6. Camellia sinensis and Osteoporosis

Osteoporosis is a very common metabolic skeletal disorder characterized by bone formation reduction (decrease in osteoblast number) and bone resorption increase (sex hormone lack). As a consequence of this disequilibrium between both processes, there is a bone mass loss and a bone tissue deterioration leading to an increase risk of fractures especially hip and vertebral fractures [100]. Osteoporosis is a multifactorial disease, with age being the most common risk. Other risk factors include environmental (i.e., alcohol consumption, smoking, vitamin D and calcium deficiencies, low physical activity), metabolic (estrogen deficiency), and genetic factors (i.e., cathepsin K, sclerostin, chloride channel 7, high-risk ethnic groups) [101]. Osteoporosis affects around 200 million people worldwide (30% of women, 12% of men) [101].

Osteoporosis *in vitro* models commonly employ mouse macrophage-like RAW264.7cell lines. Receptor activator of nuclear factor-κB ligand (RANKL) is used to differentiate macrophage cells into mature osteoclasts [68]. Moreover, for *in vivo* studies, the most common animal model is ovariectomized rats. Ovariectomized rat bone loss has many similarities to those of postmenopausal bone loss such as bone resorption increases more than bone formation and intestinal calcium absorption reduction; these related characteristics make ovariectomized rats an ideal animal model to study osteoporosis pathogenesis [102].

Green tea extract (25, 50, and 100 µg/mL, 48 h) has shown to inhibit RANKL-induced osteoclast formation in the mouse macrophage-like cell line RAW264.7 related to NFATc1, cathepsin K, C-Fos, and MMP-9 gene levels reduction [103]. Moreover, the isolated compound gallocatechin gallate (oxidation product of epigallocatechin-3-gallate) at 10 µM concentration inhibited osteoclastogenesis more potently than epigallocatechin-3-gallate through gene and protein downregulation (TRAP, c-Src, β3-Integrin, cathepsin K, and MMP-9) and master transcriptional regulators downregulation (NFATc1 and c-Fos) in RAW264.7 cells [104].

Redox imbalance is also involved in the pathogenesis of bone loss. Overproduction of ROS increases osteoclast activity and inhibits mineralization [105]. Flavones from tea have demonstrated to act as antioxidants. Particularly, epicatechin isolated from Huangshan Maofeng tea (green tea produced in Anhui province of China) has shown to protect against oxidative stress in a hydrogen peroxide-induced model on C2C12 mouse myoblast cells [106].

Moreover, *in vivo* evidence has demonstrated that tea exerts a protective effect on osteoporosis as evidenced in relevant biomarkers. Hence, green tea extracts (dose of 370 mg/kg for 13 weeks) increase cortical and trabecular bone mass in ovariectomized female Wistar rats [107]. Furthermore, green tea polyphenols supplementation (4 months) improved bone properties (alleviate bone loss and favored bone microstructure restructuring) in obese rats fed with a high fat diet and a high fat diet followed by a caloric restricted diet [108].

Patients with diabetes mellitus have low bone mass which increase fracture risk. Therefore, de Amorim et al. (2018) [104] conducted a double-blind, randomized, placebo-controlled clinical trial to evaluate the effect of green tea extract on bone mass of diabetic patients. This clinical trial revealed that those subjects with diabetes who received 1120 mg of green tea extract containing 560 mg of polyphenols/day for 20 weeks increased their bone mineral content.

## 4. Conclusion

This systematic review unified publications on the pharmacological effect (preclinical and clinical studies) of teas made from the leaves of *Camellia sinensis* and its isolated compounds on metabolic and endocrine disorders. Most pharmacological studies were *in vitro* and *in vivo* studies focused on diabetes and obesity. Clinical trials, although they have demonstrated promising results, are very limited. The most studied tea and isolated compounds have been green tea and epigallocatechin gallate, respectively. For almost each pathology, research has been focused on investigating the effect on different signaling pathways, oxidative stress, and relevant biomarkers. Among the types of teas, differences in pharmacological action may be explained by differing chemical compositions. Moreover, differences in activity for the same type of tea can be observed because chemical composition is highly influenced by the nature of the green shoots and the procedures to manufacture tea in the producing countries. Regarding clinical trials, the different doses, treatment duration, and subjects included in each study explain the differences in the activity of tea and bioactive compounds. Future research should be aimed at providing more clinical evidence on less studied pathologies such as osteoporosis, hypertension, and metabolic syndrome. Given the close relationship among all endocrine disorders, it would be of interest to find a standard dose of tea or their bioactive constituents that would be beneficial for all of them.

## Appendix A

**Table A1:** *In vitro* pharmacological studies for *Camellia sinensis.*

Disease	Extract/Isolated Compound	Experimental Model	Treatments	Major Findings	References
**Diabetes**	Amelliaone A	α-Glucosidase model	-	α-Glucosidase inhibition: IC_50_ = 10.2 µg/mL	[40]
	Arabinogalactan	Rat islet tumor RIN-5F cells	50 or 200 μg/mL, 2 h	↑ Insulin secretion	[26]
	Black and green teas	Mouse 3T3-L1 preadipocytes	10 µg/mL, 24 h	↑ SOD, CAT, and GPx activities ↓ Protein glycation ↓ α-Amylase and α-glucosidase activities	[42]
	Black tea aqueous extract	α-Glucosidase model α-Amylase model Caco-2 cells	-	↓ α-Glucosidase activity No effect on GLUT2 and SGLT1 uptake	[33]
	Black, green, and dark tea extracts	Human liver HepG2 cancer cells	-	↓ α-Glucosidase, aldose reductase, advanced glycation end-products ↑ Glucose uptake (dark tea extracts)	[109]
	Epicatechin gallate	*α*-Amylase model *α*-Glucosidase models	-	↓ *α*-Amylase activity (IC_50_ = 45.30 μg/mL) ↓ *α*-Glucosidase activity (IC_50_ = 4.03 μg/mL)	[39]
	Epigallocatechin gallate	Mouse 3T3-L1 adipocytes	20 μM, 2 h	↓ IGF-I and IGF-II stimulation	[27]
	Epigallocatechin-3-O-gallate	Rat skeletal muscle L6 cells	0, 20, 40, 50, and 60 μM, 48 h	↓ α-Glucosidase activity (IC_50_ = 19.5 μM) ↑ Glucose uptake Promotion GLUT4 translocation to plasma	[15]
	Flavanols	α-Glucosidase model	-	↓ Sucrase activity and maltase activity (EGCG IC_50_ = 32.5 and 1.3 μM, respectively)	[14]
	Flavone and flavone glycosides	α-Glucosidase model α-Amylase model	-	↓ α-Glucosidase activity (kaempferol monoglycoside IC_50_ = 40.02 μM) ↓ α-Amylase activity (kaempferol diglycoside IC_50_ = 0.09 μM)	[41]
	Green tea polyphenols Green, black, and oolong tea extracts	α-Glucosidase model	-	↓ α-Glucosidase activity (green tea polyphenols IC_50_ = 2.33 µg/mL, green tea IC_50_ = 2.82 µg/mL, black tea IC_50_ = 2.25 µg/mL, and oolong tea IC_50_ = 1.38 µg/mL)	[31]
**Diabetes** **(continued)**	Green, oolong, and black water and pomace tea extracts	Rat intestinal α-glucosidase activity	-	↓ α-glucosidase activity (tea water extract IC_50_ = 2040 µg/mL and tea pomace extract IC_50_ = 1950 µg/mL)	[32]
	Non-catechin flavonoids	Human liver HepG2 cancer cells	Insulin (5 µM) Insulin + TNF-α (30 ng/mL) Insulin + TNF-α + NCF (2000 ppm) Insulin + TNF-α + NCF (1000 ppm) Insulin + TNF-α + NCF (500 ppm) 2, 4, and 6 h	↑ TNF-α induced insulin resistance ↓ Glucose levels	[29]
	Pu-erh tea polysaccharides	α-Glucosidase model α-Amylase model	-	↓ α-glucosidase activity No α-amylase inhibition	[37]
	Qingzhuan dark tea	α-Glucosidase model	IC_50_ 2270 µg/mL for ethyl acetate fraction	↓ α-Glucosidase activity (ethyl acetate fraction, EGCG, ECG)	[110]
	Tea polysaccharides	α-Glycosidase model	-	↑ α-Glycosidase inhibitory activities (polysaccharides with 5 years aging)	[38]
**Hypertension**	Black tea aqueous extracts Thearubigin, theaflavin, catechin, epicatechin, epigallocatechin gallate, gallic acid, caffeine	Angiotensin converting enzyme model	Aqueous tea extract (15 µg/mL) Isolated compounds (37 µM)	↑ ACE inhibitory activity (Thearubigin, theaflavin, catechin)	[56]
	Black tea extract Theaflavin-3,3’-digallate	Endothelial cells from rat thoracic artery	Black tea (0.3–5 μg/mL), 30 min TF3 (0.03–0.5 μg/mL), 30 min	Endothelium dependent relaxations restored ↓ ROS production	[57]
**Metabolic Syndrome**	Green tea extract	Mouse 3T3-L1 preadipocytes	Green tea extract (0.2%–0.5%, w/v), 2 days	↓ Adipogenesis induced lipid accumulation ↓ C/EBPα and PPARγ expression	[64]
**Obesity**	Black tea theaflavins	Pancreatic lipase model	-	Pancreatic lipase inhibition Theaflavin-3,3′-digallate IC_50_ = 1.9 μM Theaflavin-3′-gallate IC_50_ = 4.2 μM Theaflavin-3-gallate IC_50_ = 3.0 μM Theaflavin IC_50_ > 10 μM	[81]
	Ethanol tea extracts	Porcine pancreatic lipase type II	5 mg/mL ethanol	Antilipase activity (IC_50_ = 500 µg/mL)	[80]
	Flavanols	Lipase model	-	↓ Lipase activity ECG (IC_50_ = 16.0 μM) CG (IC_50_ = 13.6 μM) Epiafzelechin-3-O-gallate (IC_50_ = 19.8 μM) EGCG (IC_50_ = 13.3 μM )	[14]
	Gallocatechin gallate Epigallocatechin gallate	Mouse 3T3-L1 preadipocytes	Gallates 0–20 μg/mL	Anti-adipogenic activity ↓ Intracellular lipid droplets (GCG, EGCG) ↓ PPAR γ, SREBP-1c and C/EBP α adipogenic transcription factors (GCG, EGCG) ↓ ROS levels (GCG) ↓ NF-κB activation (GCG) ↓ IL-6 production (GCG)	[73]
	Green tea catechins	Mouse 3T3-L1 preadipocytes	Green tea catechins with/without norepinephrine (0.1 or 1 μM) for 6 or 24 h	↑ Lipolysis via PKA-dependent pathway	[71]
	Green tea polyphenols Epigallocatechin-3-gallate	Mouse 3T3-L1 preadipocytes	Green tea polyphenols (1, 10, and 100 μg/mL) EGCG (6.8 μg/mL)	↓ Triglyceride accumulation ↓ Adipogenic factor C/EBPα, SREBP-1c, and PPARγ expression	[72]
	Traditional Korean Chungtaejeon	Mouse 3T3-L1 preadipocytes	Traditional Korean Chungtaejeon (250 μg/mL)	↓ Lipid accumulation ↓ PPARγ expression ↓ Adipocyte lipid-binding protein	[92]
**Osteoporosis**	Green tea extract	Mouse macrophage RAW 264.7 cells treated with RANKL (50 ng/mL)	25, 50, or 100 μg/mL for 48 h	↓ mRNA expression osteoclast-associated genes ↓ NFATc1, c-Fos, c-src and cath-K protein levels	[103]
	Gallocatechin gallate Epigallocatechin-3-gallate	Mouse macrophage RAW 264.7 cells	10 μM, 20 min	↓ RANKL-induced osteoclast differentiation ↓ F-actin ring formation ↓ Osteoclastogenesis-related marker genes and proteins expression, especially gallocatechin gallate	[104]
	Flavones	Rat osteoblastic cells C2C12 mouse myoblast cell line	From 3.125 to 50 μg/mL, 48 h	↑ Alkaline phosphatase activity (epicatechin) ↑ Hydroxyproline content (epicatechin) ↑ Area of mineralized bone nodules (epiafzelechin)	[106]

## Appendix B

**Table A2:** *In vivo* pharmacological studies for *Camellia sinensis.*

Disease	Extract/Isolated Compound	Experimental Model	Treatments	Major Findings	References
**Diabetes**	Black tea aqueous extract	GK rats	Group 1: black tea 31.3, 62.5, and 250 mg/kg Group 2: acarbose 0.1, 0.3, and 3.0 mg/kg Group 3: acarbose 0.3 mg/kg + black tea 31.3 mg/kg	↓ Plasma glucose levels	[33]
	Black tea aqueous extract	Alloxan-induced diabetic rats	Group 1: control Group 2: alloxan Group 3: black tea extract (1 mL/100 g body w/d for 10 days before alloxan injection and 35 days after alloxan injection) Group 4: black tea extract (35 days) Group 5: diabetic insulin group (twice a day/subcutaneous injection of three units of insulin)	↑ Plasma antioxidant potential ↓ Lipid peroxidation levels ↑ GSH levels	[22]
	Epigallocatechin-3-gallate	C57BL/6J mice	Group 1: low fat diet Group 2: high fat diet Group 3: high fat diet + EGCG (25 mg/kg) Group 4: high fat diet + EGCG (75 mg/kg)	↓ Plasma glucose ↓ Insulin level ↓ Advanced glycation end products	[11]
	Epigallocatechin-3-gallate	Wistar rats streptozotocin-nicotinamide-induced diabetic rats	Group 1: control Group 2: EGCG (2 mg/kg body wt) Group 3: diabetic control group Group 4: diabetic control group + EGCG 1 month	↓ Glucose, glycosylated hemoglobin, HOMA-IR and lipid profile level ↑ Insulin levels ↑ GSH levels and SOD and CAT activities	[23]
	Green tea decoctions Epigallocatechin gallate Epigallocatechin	Wistar rats	Group 1: water Group 2: green tea decoctions Group 3: EGCG, EGC	↓ SGLT-1 activity ↑ GLUT2 activity ↑ Glucose tolerance	[12]
	Green tea ethanol extracts	Sprague-Dawley rats	Group 1: hyperglycemic rats Group 2: hyperglycemic rats + tea extract 10% Group 3: hyperglycemic rats + tea extract 5% 8 weeks	↓ Serum glucose	[16]
	Green tea extract	Nematode *Caenorhabditis elegans*	0.1%, 48 h	↓ Glucose induced damage	[24]
**Diabetes** **(continued)**	Green tea extract	Rat model High sodium diet	Group 1: high sodium diet Group 2: high sodium diet + 2 g green tea extract in kg diet Group 3: high sodium diet + 4 g green tea extract in kg diet 6 weeks	↓ Insulin level and homeostatic model assessment	[13]
	Green tea polysaccharides	Kunming mice	Group 1: high fat diabetic control Group 2: rosiglitazone Groups 3, 4, and 5: green Tea polysaccharides (200, 400, and 800 mg/kgb.w. per day) 4 weeks	↓ Insulin resistance PI3K/Akt signal pathway	[10]
	Pu-erh tea and green tea	BALB/c mice	Group 1: glucose (2000 mg/kg) Group 2: glucose (2000 mg/kg) + pu-erh tea (800 mg/kg) Group 3: glucose (2000 mg/kg) + green tea (800 mg/kg) Group 4: glucose (2000 mg/kg) + EGCG (240 mg/kg) Group 5: glucose (2000 mg/kg) + EGCG (240 mg/kg) + caffeine (80 mg/kg) Group 6: caffeine (80 mg/kg)	↓ Blood glucose levels	[111]
	Pu-erh tea polysaccharides (TPS)	ICR mice	Group 1: control Group 2: acarbose (5 mg kg^−1^) Group 3: TPS (1 mg kg^−1^) Group 4: TPS (5 mg kg^−1^)	↓ Blood glucose levels	[37]
	Pu-erh tea extract	C57BL/6J mice	Group 1: normal chow diet Group 2: high fat diet Group 3: normal chow diet + pu-erh tea extract (5 mg/mL, 17 weeks) Group 4: high fat diet + pu-erh tea extract (5 mg/ml, 17 weeks)	↓ Gluconeogenesis related genes expression	[9]
	Tea polypeptides from green tea	High fat diet/streptozocin induced (30 mg/kg bw) diabetic mice	1000 mg/kg bw/day, p.o., 5 weeks	↓ Total urinary protein, creatinine, and urine nitrogen	[28]
	Tea water extract and tea pomace extract of green and black tea	Sprague-Dawley rats	Group 1: sucrose Group 2: tea extracts (0.5 g/kg body wt)	↓ Glucose level	[32]
**Hypercholes-terolemia**	Chungtaejeon aqueous extracts	Wistar rats high fat atherogenic diet (HFAD)	Group 1: normal basal diet Group 2: HFAD Group 3: 100 mg/kg day tea extract + HFAD Group 4: 200 mg/kg day tea extract + HFAD Group 5: 400 mg/kg day tea extract + HFAD	↓ LDL cholesterol ↓ Total serum cholesterol ↓ Hepatic cholesterol	[50]
	Epigallocatechin-gallate	Wistar rats Fluoride-induced oxidative stress mediated cardiotoxicity	Group 1: normal saline Group 2: EGCG (40 mg/kg BW/day) Group 3: sodium fluoride (25 mg/kg body weight/day, 4 weeks) Group 4: EGCG (40 mg/kg BW/day) + sodium fluoride (25 mg/kg body weight/day, 4 weeks)	↓ Lipid peroxidative markers ↓ Plasma total cholesterol ↓ Triglycerides ↓ Phospholipids ↓ LDL cholesterol ↑ HDL cholesterol	[48]
	Green tea ethanol extracts	Sprague-Dawley rats	Group 1: hypercholesterolemic rats Group 2: hypercholesterolemic rats + diet containing green tea extracts 5% Group 3: hypercholesterolemic rats + diet containing tea powder 10% 8 weeks	↓ LDL ↓ Triglycerides ↓ Cholesterol	[16]
	Green tea extracts	Rat model High sodium diet	Group 1: high sodium diet Group 2: high sodium diet + 2 g green tea extract in kg diet Group 3: high sodium diet + 4 g green tea extract in kg diet 6 weeks	↓ Total cholesterol, LDL, cholesterol serum concentrations	[13]
	Green tea polysaccharides	Kunming mice	Group 1: high fat diabetic control Group 2: rosiglitazone Groups 3, 4, and 5: green tea polysaccharides (200, 400, and 800 mg/kgb.w. per day) 4 weeks	↓ Total cholesterol ↓ LDL cholesterol	[47]
	Tea flavonols (“Sofu” green tea leaves and “Yabukita” tea leaves)	Mice model High cholesterol diet induced	Group 1: high cholesterol diet Group 2: high cholesterol diet + water Group 3: high cholesterol diet + “Sofu” green tea Group 4: high cholesterol diet + “Yabukita” tea	↓ Plasma oxidized LDL level	[59]
**Hypertension**	Black tea extract	Sprague-Dawley rats Angiotensin II induced	Group 1: control Group 2: angiotensin II (50 ng/kg/min) Group 3: angiotensin II + black tea extract (15 mg/kg/day, 14 days)	↑ Endothelium-dependent relaxations ↓ Endoplasmic reticulum stress markers levels ↓ ROS production	[57]
	Green tea from three cultivars “Yabukita”, “Sofu” and “Sunrouge”	Hypertensive rats High salt diet	Group 1: high salt water Group 2: high salt water + Yabukita Group 3: high salt water + Sofu Group 4: high salt water + Sunrouge	↓ Urinary NO metabolite ↑ Soluble guanyilate cyclase expression (Yabukita and Sofu)	[59]
**Metabolic Syndrome**	Green tea aqueous extract	Olanzapine induced Wistar rats	Group 1: control Group 2: olanzapine (5 mg/kg/day) Groups 3, 4, and 5: green tea aqueous extract (25, 50, and 100 mg/kg/day) + olanzapine Groups 6, 7, and 8: green tea aqueous extract (25, 50, and 100 mg/kg/day)	↓ Body weight gain ↓ Average food and water intake Improved changes in lipid profile ↓ Hyperleptinemia and hypertension	[67]
	Yellow tea	C57BL/6 male mice High fat diet	Group 1: low fat diet Group 2: high fat diet Group 3: high fat diet + 2.5% yellow tea Group 4: high fat diet + 0.5% yellow tea 12 weeks	↓ Body weight, liver weight, and adipose tissue weight ↓ Serum glucose, TC, TG, LDL-C, and HDL-C ↓ Glucose intolerance and insulin resistance	[68]
**Obesity**	Black tea and green tea decoctions	Male Wistar rats	Group 1: high fat diet Group 2: green tea decoction Group 3: black tea decoction 10 weeks	↑ Fecal triglycerides excretion ↓ Liver triglycerides ↓ Plasma triglycerides ↓ Body weight ↓ Glucose	[76]
	Decaffeinated green tea extract rich in EGCG	Male Swiss mice	Group 1: control diet + water (0.1 mL/day) Group 2: control diet + EGCG (50 mg/kg/day) Group 3: hyperlipidic diet + water Group 4: hyperlipidic diet + EGCG 16 weeks	↓ Body weight ↓ Insulin level ↓ Liver fat accumulation ↑ Glucose uptake	[90]
**Obesity (continued)**	Decaffeinated polyphenol extracts (green tea, black tea, and oolong tea)	C57BL/6J mice High fat/high sucrose	Group 1: low fat/high sucrose diet Group 2: high fat/high sucrose diet Group 3: high fat/high sucrose diet + green tea polyphenols Group 4: high fat/high sucrose diet + black tea polyphenols Group 5: high fat/high sucrose diet + oolong tea polyphenols	↓ Body weight ↓ Total visceral fat volume ↓ Liver lipid weight ↓ Food intake (green tea polyphenols)	[74]
	Decaffeinated green tea extract rich in EGCG	Swiss mice High fat diet	Group 1: control diet Group 2: high fat diet Group 3: control diet + placebo Group 4: high fat diet + placebo Group 5: control diet + EGCG Group 6: high fat diet + EGCG 8 weeks	↓ Body weights ↓ Serum triglyceride levels ↓ Adipocyte area	[91]
	Epigallocatechin 3-gallate	C57BL/6J mice	Group 1: low fat diet (negative control) Group 2: high fat diet (positive control) Group 3: high fat diet + EGCG (25 mg/kg) Group 4: high fat diet + EGCG (75 mg/kg)	↓Body weight ↓ Liver and kidney weight	[11]
	Epigallocatechin-3-gallate	C57BL/6 mice	Group 1: high fat diet Group 2: high fat diet + EGCG (20 mg/kg)	↓ Body weight ↓ Fat infiltration in liver tissue ↑ Serum lipid profiles	[93]
	Fermented green tea extract	C57BL/6 mice	Group 1: normal diet Group 2: high fat diet Group 3: high fat diet + fermented green tea extract	↓ Body weight gain ↓ Fat mass ↓ Glucose intolerance ↓ Fatty liver symptoms	[84]
	Green tea	C57BL/6J mice	Group 1: normal diet Group 2: high fat (60% energy as fat) Group 3: high fat + 0.25% (w/w) Green tea 12 weeks	↓ Body weight gain ↑ Energy expenditure ↓ Adiposity	[85]
**Obesity (continued)**	Green tea	C57BL/6J mice	Group 1: normal diet Group 2: high fat diet Group 3: high fat diet + 0.25% (w/w) green tea extract	↑ Lysophospholipids levels	[87]
	Green tea decoctions Epigallocatechin gallate Epigallocatechin	Wistar rats	Group 1: normal diet Group 2: high fat diet Group 3: high fat diet + green tea decoctions	↓ Body weight ↓ Triglycerides ↓ Cholesterol	[12]
	Green tea extract	C57BL/6J mice	Group 1: green tea extract (77 mg/g) Group 2: voluntary exercise Group 3: green tea extract + voluntary exercise	↑ Adipose tissue conversion into brown fat like adipose	[83]
	Green tea extracts	C57BL/6 mice	Group 1: control diet Group 2: high fat diet Group 3: high fat diet + 0.5% polyphenolic green tea extracts 8 weeks	↓ Adiposity ↓ High diet inflammation ↓ Adipocyte size ↓ Lipid droplet size	[86]
	Green tea extract	Sprague–Dawley rats	Group 1: normal diet control Group 2: high fat diet control Group 3: orlistat control (50 mg/kg/d + high fat diet) Group 4: green tea extract (100 mg/kg/d + high fat diet) 50 days	↓ Body weight ↓ White adipose tissue fat	[89]
	Oolong tea water extract	C57BL/6J mice	Group 1: normal diet Group 2: high fat diet Group 3/4/5: oolong tea (different storage years) 6 weeks	↓ Body weight ↓ Fat accumulation ↓ Triglyceride levels ↓ LDL cholesterol ↑ HDL cholesterol level	[75]
	Polyphenol-rich green tea extract	C57BL/6 mice	Group 1: fed a standard diet + gavage with water Group 2: standard diet + gavage with 500 mg/kg GT Group 3: HFD + gavage with water Group 4: HFD+ gavage with GT 16 weeks	↓ Body weight ↓ Body adiposity ↓ Inflammation ↑ Insulin sensitivity	[88]
**Obesity (continued)**	Polysaccharides, polyphenols and caffeine from green tea	Sprague-Dawley rats High fat rats	Groups control, polysaccharides, polyphenols, and caffeine at two doses (low and high)	↓ Body weight and fat accumulation ↑ Antioxidant levels ↓ Leptin levels ↓ Fatty acids absorption	[112]
	Pu-erh tea extract	C57BL/6J mice	Group 1: normal chow diet Group 2: high fat diet Group 3: normal diet + tea extract (5 mg/mL, 17 weeks) Group 4: high fat diet + tea extract (5 mg/mL, 17 weeks)	↓ Obesity ↓ Hepatic steatosis and liver inflammation ↓ Liver injury	[9]
	Teasaponin	High fat diet C57BL/6 male mice	High fat diet (8 weeks) + oral teasaponin (0.5%) with high diet (6 weeks)	↓ Neuroinflammation ↑ Brain derived neurotrophic factor ↑ Glucose tolerance ↓ Body weight gain	[82]
	Traditional Korean Chungtaejeon	C57BL6J-Lep ob/ob mice	Traditional Korean Chungtaejeon (200 or 400 mg/kg body weight, 10 weeks)	↓ Body weight gain ↓ Fat mass ↓ Food efficacy ratio ↓ Levels of plasma triglyceride and total cholesterol	[92]
	Water extract of white tea, yellow tea, oolong tea, green tea, white tea, and raw pu-erh tea	High fat diet induced obese mice	Group 1: untreated Group 2: atorvastatin-treated (oral daily at 10 mg/kg body weight) Group 3: green tea Group 4: yellow tea Group 5: black tea Group 6: white tea Group 7: raw pu-erh tea Group 8: oolong tea Teas: daily oral 1000 mg/kg body weight for 9 weeks	↓ Body weight ↓ White fat accumulation ↑ Energy expenditure and fatty acid oxidation (white, yellow, and oolong teas) ↓ Fatty acid synthesis (green, white, and raw pu-erh teas) Best tea: white tea	[77]
**Osteoporosis**	Green tea aqueous extract	Ovariectomized female rats	GTE (60, 120, and 370 mg/kg, 13 weeks)	↑ Bone mass ↓ Trabecular bone loss	[103]
	Green tea polyphenols	Sprague-Dawley	Group 1: high fat diet Group 2: caloric restricted diet Group 3: high fat diet + 0.5% green tea polyphenols Group 4: caloric restricted diet + 0.5% green tea polyphenols	↑ Femoral mass and strength ↑ Trabecular thickness and number ↑ Cortical thickness of tibia ↓ Trabecular separation ↓ Formation rate and eroded surface at proximal tibia ↓ Insulin-like growth factor-I and leptin	[108]

## Appendix C

**Table A3:** Clinical trials for *Camellia sinensis.*

Study (Author, Year, Country)	Study Design	Sample Size	Population	Type of Plant	Intervention	Duration of Treatment	Results
**DIABETES**							
Alves Ferreira et al., 2017 [43] Brazil	Randomized, double-blind, placebo-controlled study	120	Women (20–45 years) abnormal glucose values	Green tea capsules	Group 1: control (cellulose) Group 2: green tea (1 g) Group 3: metformin (1 g) Group 4: green tea (1 g) + metformin (1 g)	12 weeks	Improving glycemic and lipid profile ↓ Fasting glucose ↓ Total cholesterol and LDL
Lasaite et al., 2014 [113] Lithuania	Randomized double-blind placebo-controlled study	56	Patients (37–78 years) with diabetes mellitus type II and diabetic retinopathy, nephropathy or neuropathy	Green tea extract	Group 1: placebo Group 2: *Gingko biloba* dry extract Group 3: green tea extract For extracts: one capsule twice a day (9 months) and one capsule three times a day (9 months)	18 months	No statistically significant differences in HbA1c level, antioxidant state, and psychological data
Mahmoud et al., 2016 [44] Kuwait	Randomly assigned	34	Male and female type 2 diabetics	Black tea infusions	Group 1: three cups black tea daily (600 mL) Group 2: one cup black tea daily (200 mL)	12 weeks	↓ HbA1c levels ↑ Regulatory T cells ↓ Pro-inflammatory
Spadiene et al., 2014 [45] Lithuania	Randomized, double-blind, placebo-controlled study	45	Patients (35-80 years) with diabetes mellitus type II and diabetic retinopathy, nephropathy or neuropathy	Green tea extract	Group 1: green tea extract Group 2: placebo	9–18 months	↓ Lipid peroxidation
Vaz et al., 2018 [46] Brazil	Randomized, double-blind, placebo-controlled study	60	Patients with diabetes	Green tea extract	Group 1: green tea extract (two capsules/day, containing 560 mg of polyphenols/each) Group 2: cellulose (two capsules/day)	20 weeks	No effect on total antioxidant capacity, glycemic control markers, and renal function ↑ SOD activity
**HYPERCHOLESTEROLEMIA**							
Imbe et al., 2016 [53] Japan	Randomized, double-blind, placebo-controlled trial	155	Healthy volunteers High LDL cholesterol levels Aged 20–80 years	“Benifuuki” green tea	Group 1: “Benifuuki” Group 2: “Yabukita” Group 3: barley infusion drinker	12 weeks	↓ LDL cholesterol levels ↓ Lectin-like oxidized LDL receptor-1 containing LAB level
Orem et al., 2017 [51] Canada	Randomized, double-blind, placebo-controlled study	125	Subjects 25–60 years hypercholesterolemia	Black tea	Group 1: placebo Group 2: instant black tea Group 3: functional black tea	4 weeks	Functional black tea: ↓ Total cholesterol ↓ LDL ↓ Oxidative stress index ↑ Total antioxidant status
Troup et al., 2015 [52] United States	Randomized, double-blind, crossover trial	57	45–65 years, hypercholesterolemia	Black tea	Group 1: controlled low flavonoid diet plus five cups per day of black tea Group 2: Placebo	4 weeks	↓ LDL/HDL ratio ↓ Total cholesterol
**HYPERTENSION**							
Alkerwi et al., 2015 [60] Luxembourg	National cross-sectional stratified sample	1352	18–69 years	Tea	Group 1: nonconsumers Group 2: ≤ 3-dL/d consumers (tea/coffee) Group 3: > 3-dL/d consumers (tea/coffee)	-	↓ Systolic BP and pulse pressure
**METABOLIC SYNDROME**							
Yang et al., 2014 [64] China	-	134	Metabolic syndrome	Green tea extract	Group 1: green tea extract (500 mg). Two capsules/time/day Group 2: control (water)	45 days	↑ Adiponectin serum concentrations ↓ Visfatin levels
**OBESITY**							
Chen et al., 2016 [94] Taiwan	Randomized, double-blind trial	102	Women BMI ≥ 27 kg/m^2^ Waist circumference ≥ 80 cm	EGCG	Group 1: placebo Group 2: high dose green tea	12 weeks	↓ Weight ↓ Waist circumference ↓ Total cholesterol and LDL plasma levels
Dostal et al., 2016 [96] USA	Randomized, double-blind, placebo-controlled clinical trial	937	Postmenopausal women aged 50–70 with high breast density and overweight/obese	Green tea extract	Group 1: placebo Group 2: EGCG (843 mg), four capsules daily	12 months	No ↓ adiposity No improvements in BMI ↓ Tissue fat and gynoid fat
Huang et al., 2018 [95] Taiwan	Randomized, double-blind, crossover, placebo-controlled	90	Women (18 - 65 years) BMI ≥ 27 kg/m^2^ LDL-C ≥ 130 mg/dL	Green tea extract	Group 1: placebo Group 2: one capsule 30 min after meal, three times a day, green tea extract	6 weeks	↑ Leptin ↓ LDL
Janssens et al., 2015 [98] The Netherlands	Randomized, placebo-controlled, single-blind design	60	Caucasian men and women with body mass index from 18 kg/m², age: 18–50	Green tea extract	Group 1: placebo Group 2: green tea (capsules > 0.06 g EGCG and 0.03–0.05 g caffeine)	12 weeks	No effect on fecal energy content, fecal fat content, resting energy expenditure, respiratory quotient, and body composition
Mielgo-Ayuso et al., 2014 [97] Spain	Randomized, double-blind, parallel design	83	Obese (30 kg/m^2^. BMI, 40 kg/m^2^) premenopausal women	EGCG	Group 1: placebo (lactose) Group 2: EGCG (300 mg/d)	12 weeks	No changes in body weight No changes in adiposity
Nicoletti et al., 2019 [99] Brazil	Longitudinal interventional study	11	Women (18–60 years) (BMI) > 40 kg/m^2^	EGCG	Group 1: eutrophic women Group 2: decaffeinated green tea capsules with 450.7 mg of EGCG, two capsules/day	8 weeks	↑ RICTOR ↑ HIF1-α expression
**OSTEOPOROSIS**							
Amorim et al., 2018 [114] Brazil	Double-blind, randomized, controlled clinical trial	35	≥ 18 years old Diabetes for more than 5 years.	Green tea extract	Group 1: cellulose Group 2: 1120 mg of green tea extract contains 560 mg of polyphenols/day	10 and 20 weeks	↑ Bone mineral content
